# Nanospider-Generated Polyamide 6 Scaffolds Nanostructured with Graphene Oxide for Enhanced Cell Adhesion and Tissue Development

**DOI:** 10.3390/ijms27135826

**Published:** 2026-06-27

**Authors:** Michał Pruchniewski, Damian Nakonieczny, Malwina Sosnowska, Totka Bakalova, Petr Louda, Agnieszka Ostrowska, Patryk Pokorski, Zofia Nowak, Ewa Sawosz, Barbara Strojny-Cieślak

**Affiliations:** 1 Department of Nanobiotechnology, Institute of Biology, Warsaw University of Life Sciences, Ciszewskiego 8 Str., 02-786 Warsaw, Poland; michal_pruchniewski@sggw.edu.pl (M.P.);; 2 Department of Materials, Faculty of Mechanical Engineering, Technical University of Liberec, Studentska 2 Str., 461 17 Liberec, Czech Republic; 3 Faculty of Technology and Education, Koszalin University of Technology, Kwiatkowskiego 6e Str., 75-343 Koszalin, Poland; 4 Department of Technique and Food Product Development, Institute of Human Nutrition Sciences, Warsaw University of Life Sciences, Nowoursynowska 159c Str., 02-776 Warsaw, Poland

**Keywords:** polyamide-6, graphene oxide, nanospider, cell adhesion, mechanotransduction, tissue regeneration

## Abstract

Graphene oxide (GO)-based nanostructured biomaterials have emerged as promising platforms for tissue engineering due to their novel biointeractive properties. In this study, we developed polyamide 6 (PA6) scaffolds by electrospinning using the Nanospider technique. Unlike conventional laboratory-scale electrospinning systems, Nanospider™ employs a wire-based electrode coated with a thin layer of polymer solution, from which nanofibers are continuously generated under a high-voltage electric field, enabling the large-scale fabrication of scaffolds. The scaffolds were then nanostructured with GO to investigate the effect of surface modification on their physicochemical properties, and biological responses. Surface characterization demonstrated that GO incorporation altered the microtexture of PA6 scaffolds, leading to changes in topographical parameters and surface morphology. In vitro studies performed using human stromal HS-5 cells confirmed high cytocompatibility of both GO nanofilms and PA6-GO composites, with preserved metabolic activity and enhanced cell adhesion. Scanning electron microscopy revealed improved spreading, elongated morphology, and increased filopodia formation on GO-modified scaffolds. Gene expression analyses indicated modulation of mechanotransduction- and adhesion-related pathways, including differential regulation of *FN1*, *FAK*, and integrin-associated genes, suggesting that GO nanostructuring influences early cell–material interactions through combined effects on surface architecture and chemistry. *Ex vivo* studies using embryonic tissues derived from chicken embryo *Gallus gallus* demonstrated effective colonization of connective, cartilage, and bone tissues on GO-modified scaffolds. Collectively, these findings demonstrate that GO nanostructuring of electrospun PA6 scaffolds improves biointerface formation, supports mechanobiological adaptation, and promotes tissue development, highlighting the potential for regenerative medicine.

## 1. Introduction

Biomimetic hybrid biomaterials constitute an advanced category of engineered constructs in tissue engineering, characterized by the strategic integration of diverse fabrication techniques and material constituents to optimize their therapeutic efficacy. This multidisciplinary approach facilitates the synergistic combination of materials properties, enabling the exploitation of their inherent advantages while minimizing individual limitations. The rational and precise design of these hybrid systems aims to replicate the complex microarchitectural and functional features of native tissues at the molecular and cellular level, which will allow for increased precision and effectiveness of tissue regeneration strategies. One proposed approach involves the integration of nanotechnology with biomaterials science through the development of polymer matrices embedded with nanostructures, such as graphene oxide, to enhance their physicochemical characteristics and biological performance.

Graphene oxide (GO) is a two-dimensional nanostructure consisting of a monolayer of sp^2^-hybridized carbon atoms arranged in a hexagonal honeycomb lattice [1]. Its basal plane and edges are extensively functionalized with oxygen-containing groups [2,3] such as hydroxyl, epoxy, and carboxyl moieties, which enhance GO’s dispersibility [4], facilitate chemical modifications, and improve biocompatibility relative to pristine graphene [5]. In the biomedical context, GO has been demonstrated to enhance cellular responses by promoting increased cell adhesion [6,7], proliferation [8,9], and migration [10]. At the molecular level, GO can modulate gene expression profiles and influence secretory pathways, thereby affecting cell behavior and function [11,12]. These multifaceted biological effects, combined with its tunable physicochemical properties, position GO as a highly promising nanomaterial for applications in tissue engineering.

A key challenge in the clinical application of GO nanocoatings is their intrinsic brittleness and mechanical fragility, which predispose them to delamination and deformation, thereby compromising their physicochemical stability and biocompatibility [13,14,15]. Deposition of GO onto polymer substrates offers a strategic approach to mitigate these limitations. This methodology enhances processability, handling and safety by anchoring GO nanolayers onto a stable, biocompatible matrix, reducing aggregation and transfer risks, and minimizing unintended GO nanoflakes release. Furthermore, interfacial interactions between GO and the polymer matrix can induce nanostructural modifications, enabling the precise tuning of surface topography, mechanical properties, and biocompatibility for optimized biomedical performance [16,17]. An illustrative example of such a polymer stabilizing GO is electrospun polyamide 6.

Polyamide 6 (PA6) is an aliphatic synthetic polyamide synthesized via the ring-opening polymerization of ε-caprolactam, resulting in a semi-crystalline thermoplastic polymer characterized by repeating amide linkages [18]. Its molecular architecture confers high tensile strength, toughness, elasticity, and resistance to chemicals, abrasion, and fatigue [19]. Biomedically, PA6 exhibits favorable biocompatibility, making it suitable for sutures, implants, and tissue scaffolds; its non-degradable nature ensures long-term structural stability. PA6 can be processed through electrospinning techniques, such as Nanospider technology [20]. Nanospider is an advanced electrospinning technique that produces ultrafine fibers from various polymers by utilizing a high-voltage electrostatic field to generate a charged polymer jet from a Taylor cone [21]. Unlike traditional electrospinning, it employs a rotating roller immersed in the polymer solution, enabling the formation of multiple Taylor cones along its surface [22]. This design significantly enhances scalability and production throughput compared to conventional electrospinning, while still maintaining the biomimetic properties of the resulting nanofibrous mats. However, the inherent bioinertness of electrospun PA6 scaffolds constrains cellular adhesion and proliferation despite their biomimetic fibrous architecture, thereby necessitating surface modifications or coatings to improve biocompatibility and facilitate biological integration. The literature indicates that the incorporation of GO into electrospun polymer structures enhances cell adhesion [23], promotes osteogenic differentiation [24], and increases mineralization [25] in comparison to pure polymer scaffolds. Specifically, the integration of GO nanocoating into the polymer matrix may enhance cellular mechanotransduction pathways by augmenting surface roughness and providing bioactive cues.

In this study, we present a bioactive and biomimetic hybrid material comprising PA6 electrospun via the Nanospider technique, nanostructured with GO. The investigation elucidates the effects of polymer nanostructuring on key physicochemical parameters, including surface texture and morphological characteristics. Biocompatibility assessments confirmed the favorable cellular response to GO nanocoatings as well as to nanostructured PA6-GO composites. Furthermore, the study highlights the influence of polymer nanostructuring on mechanotransduction processes, evidenced by alterations in cellular ultrastructure and the differential expression of adhesion-related genes. Additionally, long-term studies on the colonization of scaffolds by embryonic cells and tissues were conducted, supporting the scaffold’s suitability for tissue regeneration and integration over extended periods, indicating potential implications for tissue engineering and regenerative medicine applications.

## 2. Results

### 2.1. GO Characterization

**Figure 1:**
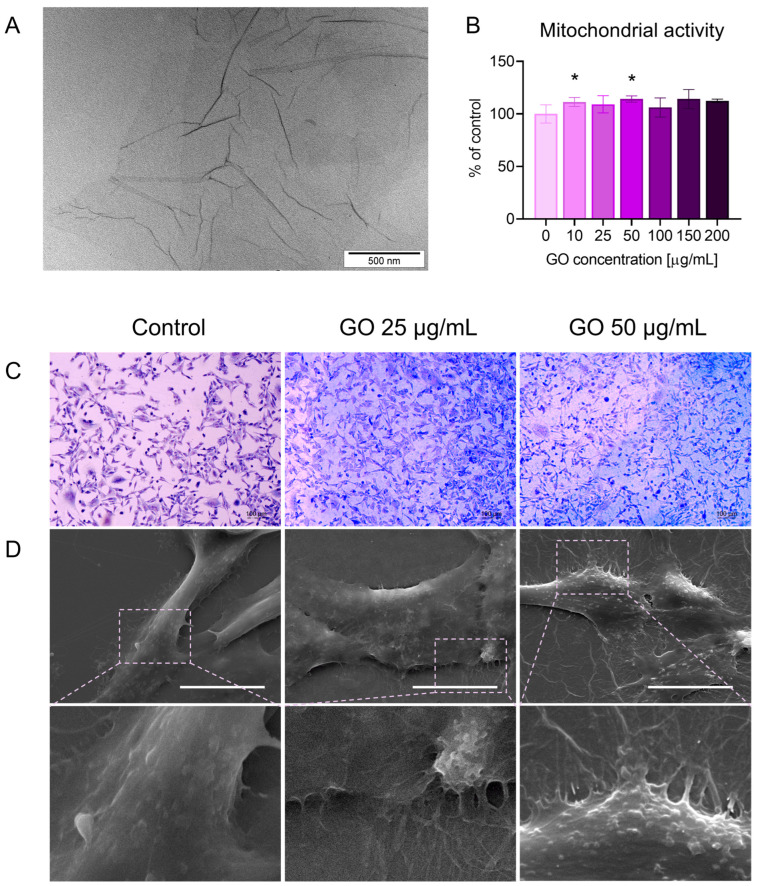
Biocompatibility assessment of graphene oxide (GO) nanolayers on the HS-5 cell line: (**A**) Morphology of GO nanosheets observed by transmission electron microscopy (TEM); (**B**) Cells viability after 24 h incubation on GO nanolayers assessed by the MTT assay; results are expressed as a percentage of the control (mean ± SD; *n* = 4); statistically significant differences between the control and study groups are marked as * *p* < 0.0332; (**C**) Visualization of cells on GO nanolayers using optical microscopy after May-Grünwald-Giemsa (MGG) staining; scale: 100 µm; (**D**) Cell morphology on GO nanolayers observed by SEM; scale: 20 µm.

For biocompatibility evaluation, GO films were fabricated from GO flakes, whose morphology was characterized by TEM (Figure 1A). GO-based nanofilms did not exhibit any cytotoxicity toward HS-5 cells, as assessed by mitochondrial dehydrogenase activity in the MTT assay after 24 h (Figure 1B). Moreover, the nanofilm promoted cell growth, with statistically significant differences observed at low to medium concentrations.

Assessment of cell morphology after MGG staining (Figure 1C) revealed that cells cultured on GO nanofilms at concentrations of 25 and 50 mg/L exhibited a morphology similar to that of the control group; however, their culture density was higher. A subset of cells cultured at the higher GO concentration displayed elongated filopodia connecting adjacent cells on the film, in comparison to the control group seeded directly on the culture plate.

The samples’ visualization at high resolution by SEM (Figure 1D) confirmed that HS-5 cells’ morphology appeared normal; however, it was observed that cells cultured on GO nanofilms were more flattened on the surface compared to the control group (bare coverslip). Additionally, in the case of the GO nanofilm at a concentration of 50 mg/L, a greater number of cytoplasmic protrusions was observed compared to the 25 mg/L group. Longer cytoplasmic extensions were also visible in the experimental groups than in the control group, which was in accordance with the observation after MGG staining; similarly, connections between cells were observed in the form of connections formed by cytoplasmic protrusions, and the general density on the GO nanofilms was increased.

### 2.2. PA6 and PA6 + GO Composite Characterization

**Figure 2:**
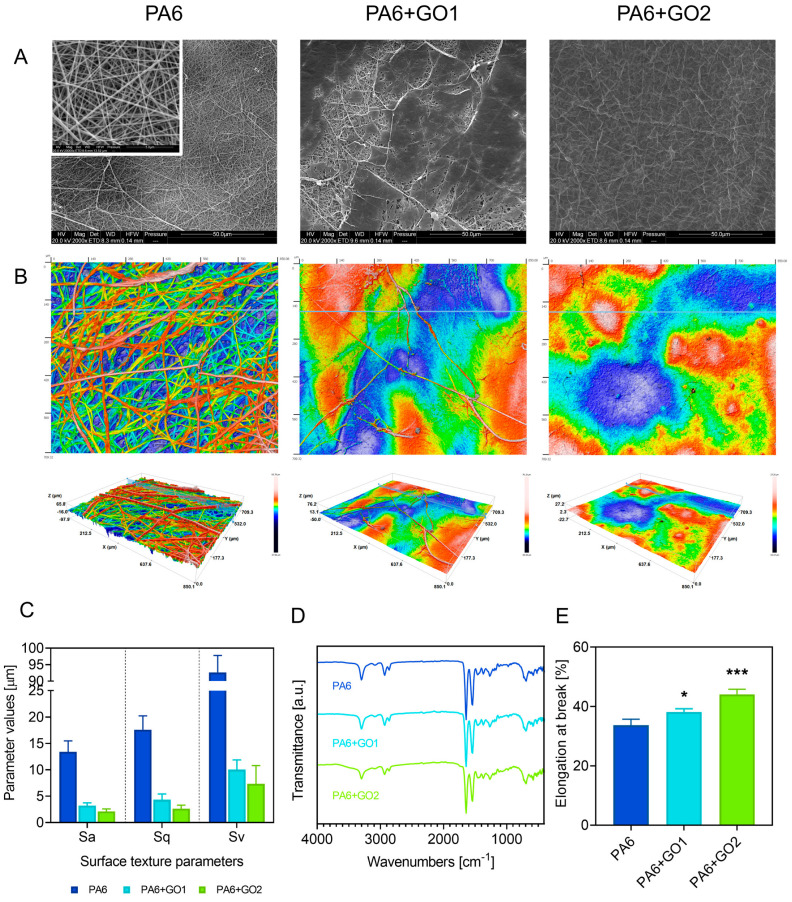
Characterization of polyamide 6 (PA6) scaffolds before and after nanostructuring with graphene oxide (GO) by the shake−out method (PA6 + GO1) and direct deposition method (PA6 + GO2): (**A**) Visualization of the nonwovens using a scanning electron microscope (SEM); scales: 50 µm and 5 µm (zoom); (**B**) 2D and 3D visualization of the surface roughness using a Sensofar confocal profilometer; size of the assessed area: 709.32 µm × 850.08 µm (2D) × height (3D); the blue line indicates the region used for surface analysis; (**C**) Height parameters of surface roughness: Sa (arithmetical mean height), Sq (root mean square height), Sv (maximum pit depth); results are presented as means ± SD (*n* = 3); (**D**) FT−IR spectra for samples before and after nanostructuring. (**E**) Mechanical properties of scaffolds expressed as elongation at break; statistically significant differences between the PA6 and GO-modified PA6 are marked as * *p* < 0.0332, and *** *p* < 0.0002.

SEM analysis confirmed the high efficacy of PA6 fabrication via the Nanospider technique (Figure 2A). The polymer fibers exhibited well-defined morphology, devoid of artifacts, with a consistent diameter of approximately 200 nm throughout their entire length. The resulting nonwoven fabric demonstrated porosity and maintained structural uniformity. Nanostructuring of PA6 through the shaking method yielded partial and uneven coverage of the polymer surface with GO, whereas direct deposition of nanoflakes proved significantly more effective in achieving uniform modification. In this approach, GO was homogeneously distributed and coated onto the polymer matrix, resulting in a more consistent surface modification. The incorporation of GO-induced surface structuring of the PA6 fibers, with the fiber morphology guiding the pattern and uniformity of the nanolayer deposition.

Confocal microscopy analysis utilizing the Sensofar system (Figure 2B) revealed a statistically significant reduction in surface roughness parameters, including Sa (arithmetical mean height), Sq (root mean square height), Sv (maximum pit depth), for the nanostructured nonwoven fabrics compared to the unmodified polymer matrix (Figure 2C). These quantitative surface characterization metrics indicate increased surface uniformity and modification of the topographical features attributable to the nanostructuring process. This analysis is consistent with the SEM visualization, supporting the conclusion that GO integration contributes to surface smoothing. The combined surface analytical data suggest that nanostructuring and GO addition processes synergistically enhance surface homogeneity, thereby optimizing the surface topography for potential functional applications.

The FT-IR analysis revealed no significant differences between the fibers before and after nanostructurization, indicating that the primary chemical structure of the PA6 polymer remained unchanged during the modification process (Figure 2D). The characteristic peaks for PA6 were clearly evident, including the amide I band around 1650 cm^−1^, the amide II band near 1550 cm^−1^, and the N–H stretching vibrations around 3300 cm^−1^. These peaks confirm the presence of the typical polyamide functional groups and suggest that the nanostructurization process did not alter the fundamental chemical composition of the polymer fibers.

Additionally, the mechanical properties of PA6 were evaluated prior to and following GO nanostructuring by measuring the elongation at break (Figure 2E). The elongation at break values were 33.75%, 38.16%, and 44.09% for PA6, PA + GO1, and PA + GO2, respectively. These results indicate a significant enhancement in the mechanical performance of PA6 after nanostructuring compared to the pure polymer.

### 2.3. Biological Properties of the PA6 + GO Composite

Although GO possesses inherently advantageous biological properties, the incorporation of graphene oxide into biomaterials necessitates rigorous re-evaluation of their biological performance. This is essential because the biological interactions and effects can vary significantly depending on the specific material formulation, processing methods, and environmental conditions.

**Figure 3:**
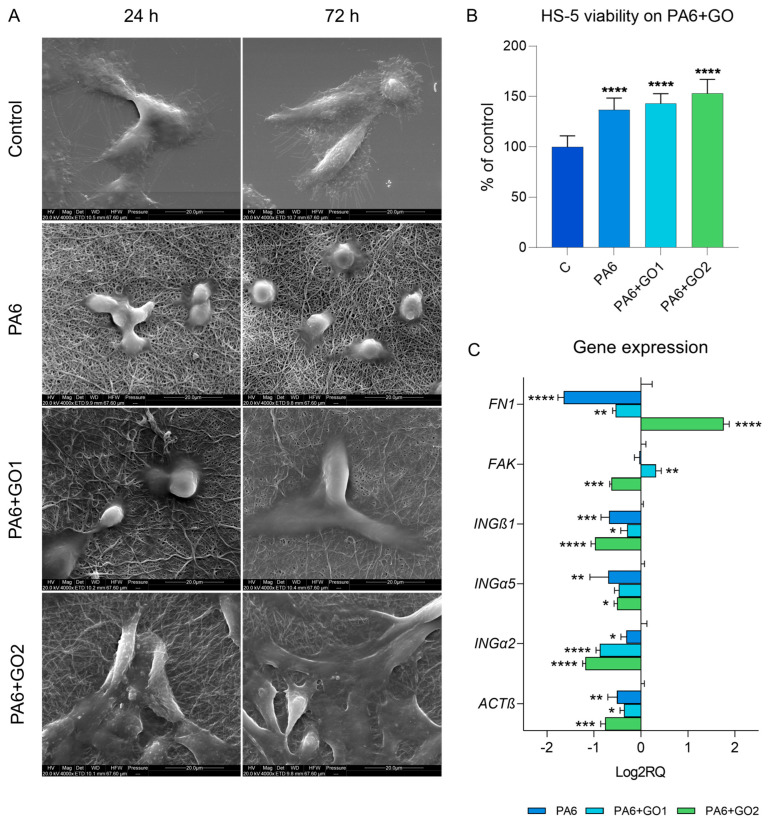
Evaluation of the bioactivity of polyamide 6 (PA6) scaffolds nanostructured with graphene oxide (GO), prepared via agitation method (PA6−GO1) or direct deposition (PA6−GO2), using human mesenchymal stromal cells (HS−5): (**A**) Visualization of cell colonization on the scaffolds at 24 h and 72 h using scanning electron microscopy (SEM); scale bar: 20 µm; (**B**) Assessment of scaffold biocompatibility after 24 h of cell seeding using the PrestoBlue viability assay; results expressed as a percentage of control values (mean ± SD; *n* = 3); (**C**) Analysis of the expression levels of genes associated with cell adhesion and mechanotransduction in cells after 24 h, determined by quantitative real−time PCR (qRT−PCR); results are presented as log2 relative quantification (log2RQ) ± SD (*n* = 3); statistically significant differences between the control and study groups are marked as * *p* < 0.0332, ** *p* < 0.0021, *** *p* < 0.0002, and **** *p* < 0.0001.

SEM analysis confirmed the effective colonization of PA6 scaffolds by HS-5 cells irrespective of GO nanostructuring at both 24 h and 72 h time points (Figure 3A). At 24 h, cells seeded on PA6 and PA6 + GO1 exhibited a predominantly rounded morphology, whereas cells on PA6 + GO2 displayed a spindle-shaped and flattened morphology that resembled that of control cells, characterized by the formation of branched filopodia. After 72 h, cells on pure PA6 largely retained their spherical shape, while those on PA6 + GO1 adopted a spindle-like morphology. Conversely, cells on PA6 + GO2 appeared more extensively flattened, with increased anchorage to the scaffold surface and a higher cell density. No evidence of cytotoxicity, such as apoptotic body formation or membrane perforation, was observed on any of the scaffolds at either time point, as confirmed by viability assessment via the PrestoBlue assay (Figure 3B). Importantly, cells cultured on the scaffolds showed increased viability compared to control cells, exceeding 120% of the viability of control cells, confirming the safety and biotolerance of the cell scaffolds in vitro.

Quantitative analysis via real-time PCR revealed significant alterations in gene expression at the mRNA level in cells cultured on various scaffold substrates (Figure 3C). Specifically, genes implicated in cellular adhesion and mechanotransduction pathways were evaluated, including fibronectin (*FN1*), focal adhesion kinase (*FAK*), integrin subunits β1, α5, and α2 (*INGs*), as well as β-actin (*ACTβ*). The results demonstrated a marked upregulation of *FN1* expression in cells maintained on PA6-GO2, while a significant downregulation was observed in cells cultured on the remaining two scaffold variants relative to the control group. In addition, *FAK* expression was up-regulated in cells maintained on PA6 + GO1, with further increased expression observed following culture on PA6 + GO2 scaffold. A consistent trend was observed across the *INGs* subunits and *ACTβ*, characterized by a generalized downregulation of gene expression across all PA6-based scaffolds, irrespective of their nanostructuring modifications, compared to control. This downregulation was most pronounced in cells cultured on PA6-GO1 and PA6 + GO2 scaffolds compared to neat PA6.

**Figure 4:**
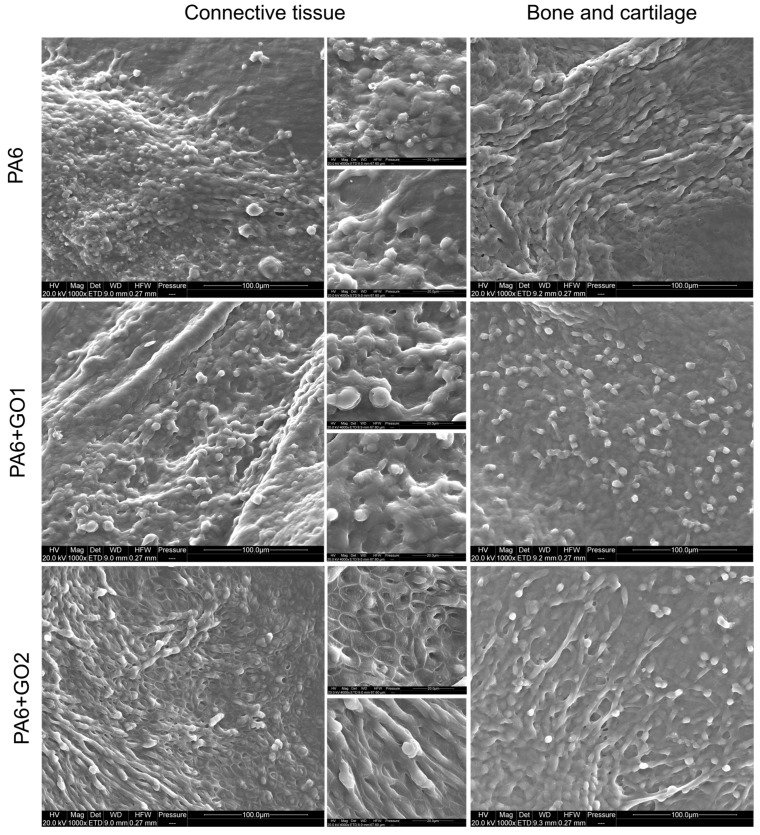
Assessment of the interactions between polyamide 6 (PA6) cellular scaffolds nanostructured with graphene oxide (GO) following different preparation methods—shaking (PA6-GO1) and direct deposition (PA6-GO2)—and connective, osseous, and cartilaginous tissues derived from 9-day-old *Gallus gallus* embryos. Visualization was conducted after a 7-day incubation period using scanning electron microscopy (SEM). Scale bars: 100 µm (overview), 20 µm (high-magnification images).

A comprehensive long-term assessment of the interaction between PA6 scaffolds and embryonic tissues of *Gallus gallus* was conducted utilizing SEM. This technique facilitated the acquisition of high-resolution, detailed images of the scaffold–tissue interface, enabling the examination of morphological alterations at both cellular and tissue levels over an extended 7-day incubation period (Figure 4). The analysis revealed active tissue development and the presence of newly forming cells embedded within the scaffolds across different tissue types, including connective tissue, bone, and cartilage, independent of the nanostructuring of the scaffolds with GO.

At the cellular level, embryonic connective tissue cultured on PA6 and PA6-GO1 scaffolds predominantly exhibited cells with a spherical, rounded morphology. This shape is characteristic of less differentiated or more primitive cells, often linked to a migratory or undifferentiated state. Such rounded cell forms might be related to limited spreading and may indicate that cells remain in an early stage of development within these scaffolds. Conversely, on PA6-GO2 scaffolds, more dynamic tissue formation was observed, marked by the emergence of diverse cellular populations with varied morphologies. These included cells with flattened, elongated shapes reminiscent of fibroblasts, as well as polygonal cells displaying evident cell–cell contacts, similar to epithelial phenotypes. Focusing specifically on bone and cartilage tissues, distinct cellular behaviors were noted depending on the scaffold type. On PA6-GO1 scaffolds, cells predominantly retained a spherical shape, typically may be associated with chondroprogenitors or early chondrogenic stages. In contrast, on PA6 and PA6-GO2 scaffolds, a combination of spherical and elongated cell morphologies was observed, with elongated cells often aligning along specific directions and forming organized growth patterns. While the observed morphological features indicate enhanced tissue development, precise identification of the participating cell populations and the exact stage of tissue maturation would require further advanced analyses.

## 3. Discussion

GO is broadly recognized as a biocompatible nanomaterial in contact with cellular systems. Nonetheless, the determination of an appropriate dosage is critical, as excessive or improper concentrations of GO may elicit cytotoxic effects. The cytotoxicity is hypothesized to result from physical interactions between GO nanosheets and cell membranes, including membrane disruption and lipid bilayer reorganization [26,27]. Additionally, GO can induce oxidative stress through the generation of reactive oxygen species (ROS) [28,29] and may accumulate within the membrane matrix [30,31], leading to structural integrity compromise and functional impairments of cellular components. Several studies have documented the alterations in cellular metabolic activity and morphology associated with high concentrations of GO [32,33,34], which must be carefully considered, particularly in the context of biomedical applications. A comprehensive understanding of the underlying mechanisms of GO-induced cytotoxicity is essential, as it provides critical insights for mitigating adverse effects and promoting the elicitation of targeted cellular responses.

Our recent studies [35] have demonstrated that GO in the form of a hydrocolloid at elevated concentrations can diminish mitochondrial activity, induce disintegration of the cell membrane, and promote reorganization of the actin cytoskeleton. In contrast, GO applied as a nanofilm does not exhibit these cytotoxic effects; instead, it facilitates cell adhesion by promoting the formation of filopodia networks. Importantly, GO has the potential to augment the bioactivity of raw materials commonly employed in biomaterial design and to reduce the cytotoxicity of other materials within composite systems containing GO. Single-layer GO nanoflakes have been shown to enhance the viability and proliferation of human bone marrow-derived mesenchymal stem cells encapsulated within alginate microgels [9]. However, this advantageous effect diminishes with increasing GO concentration and prolonged exposure duration. Furthermore, GO in the form of a nanofilm has the capacity to mitigate the cytotoxic effects of potentially cytotoxic compounds, such as silver nanoparticles, while preserving their inherent antibacterial properties [36]. Another example is the enhancement of alloy biocompatibility through the incorporation of GO, which contributes to increased material stability and consequently mitigates issues related to pH elevation and ion release [37].

In this study, we demonstrated the sustained metabolic activity of cells cultured on GO nanofilms, along with their normal morphology and the formation of stable anchoring structures to the GO layer via filopodia (Figure 1). GO can facilitate the formation of a stable biointerface and support the stable anchoring of cells on the biomaterial through the extension of filopodia [38,39]. For instance, Liu et al. [39] observed morphological alterations in bone marrow mesenchymal stem cells cultured on GO-containing scaffolds. Cells exhibited enhanced spreading and formed long cytoplasmic protrusions resembling pseudopodia, indicative of active interaction with the material. These protrusions were accompanied by visible reorganization of the actin cytoskeleton, suggesting enhanced mechanical stability and adhesion. Authors also reported increased expression of adhesion-related proteins, such as vinculin. GO and other oxidized graphene family nanomaterials (GFNs) can promote filopodia formation by enhancing the activity of the GTPase Cdc42, which is involved in cytoskeletal reorganization, and by inhibiting Rho-associated kinase (ROCK), a key regulator of actin contractility [40]. Furthermore, the method of GO surface preparation may influence cell adhesion, as cells on fully coated GO surfaces tend to spread extensively and exhibit an elongated morphology in all directions. In contrast, on partially coated GO substrates, cells preferentially adhere to the nanomaterial-coated regions, indicating that GO may also play a role in directing the orientation of cell adhesion [6].

Although GO possesses inherently advantageous biological properties, the incorporation of GO into biomaterials necessitates rigorous re-evaluation of their biological performance. This is essential because the biological interactions and effects can vary significantly depending on the specific material formulation, processing methods, and environmental conditions. SEM visualization demonstrated that cells cultured on the glass control surface exhibited a well-spread morphology characteristic of rigid two-dimensional substrates (Figure 3A). However, micro- and nanostructuring the cell growth surface can mitigate the effects of a stiff substrate by decreasing cellular stiffness and restricting the formation of adhesion foci [41]. Cells on neat PA6 showed more restrained early spreading, with a higher proportion of rounded morphologies and limited protrusion development (Figure 3). This phenomenon is consistent with a discontinuous adhesion landscape characteristic of fibrous and porous polymeric scaffolds, wherein cell anchorage occurs at discrete contact points rather than over a continuous surface. Excessively high porosity levels can reduce the available fiber–cell contact surface area, thereby impeding the establishment of robust cellular adhesion [42]. This problem may result from apparent difficulties in cell recognition of the substrate, as well as from differences in the distribution of traction force, which consequently influences cytoskeletal reorganization and filopodia dynamics [43,44]. In contrast, modification of the scaffold surface in PA6-GO composites resulted in enhanced cell adhesion, as the nanostructured scaffolds displayed a topography more closely resembling in vivo tissue architecture, characterized by a rough, complex, and fibrous structure. PA6 + GO2 promoted a more elongated, spindle-shaped phenotype with pronounced cellular protrusions observed even after 24 h, which became more prominent after 72 h. In contrast, PA6 + GO1 also facilitated cell spreading over time, characterized by frequent cell–fiber interactions; however, such features were predominantly evident after 72 h. This difference may be attributed to the less homogeneous surface texture of PA6 + GO1 compared to PA6 + GO2. These observations are consistent with existing literature demonstrating that surface modification of fibrous polymer scaffolds with carbon nanostructures can influence early cell attachment and cytoskeletal organization without compromising cytocompatibility. For instance, the functionalization of electrospun poly(lactic-co-glycolic acid) (PLGA) scaffolds with GO and hydroxyapatite (HA) has been shown to enhance cell spreading and induce alterations in the cytoskeletal architecture of MC3T3-E1 osteoblast precursor cells [25]. Consequently, this modification led to increased alkaline phosphatase (ALP) activity and promoted osteomineralization in vitro [25]. Additionally, a positive impact on cell spreading, along with increased calcium deposition and upregulation of osteogenic markers such as *RUNX2* and *osteopontin*, has been observed in mesenchymal stem cells and osteosarcoma cells cultured on reduced GO-containing polycaprolactone (PCL) fibrous scaffolds [45]. Furthermore, electrospun PLGA/silk fiber scaffolds incorporating GO have been demonstrated to facilitate mesenchymal cell proliferation and growth, concurrently promoting osteoclastogenic activity and enhancing calcium mineralization [46].

Importantly, the observed differences are consistent with the surface and textural characteristics of the scaffolds (Figure 2). Confocal topography maps (Figure 2B) and texture analysis (Figure 2C) reveal that GO-modified PA6 surfaces exhibit altered textural features and significantly reduced height-related descriptors (Sa, Sq, Sv) compared to pristine PA6. The transition from a more heterogeneous, uneven landscape to a relatively more uniform height distribution likely facilitates more stable protrusion anchoring and enhances cell attachment to the fibers, particularly during the early adhesion phase when cells interact with the substrate through adhesion-associated proteins. Collectively, these findings suggest that GO nanostructuring influences early cell morphology through combined modifications in nano- and microtexture as well as interface chemistry, thereby promoting a conducive cellular microenvironment.

The PrestoBlue assay conducted after 24 h indicated that HS-5 cells retained high metabolic activity across all PA6-based scaffolds (Figure 3B). Importantly, the signals measured on all scaffolds exceeded those observed for the glass control, indicating either a higher number of viable cells, increased metabolic activity per cell, or a combination of both. These findings, in conjunction with existing literature, corroborate the cytocompatibility of PA6 [47]. Surface modifications of PA6 have the potential to enhance metabolic activity and cell adhesion, thereby providing a foundation for tissue development, especially following optimization of the scaffold’s surface microstructure or chemical composition [48,49].

To investigate the molecular aspects of the early interface response, we quantified the expression of genes associated with adhesion and mechanotransduction after 24 h (Figure 3C). *FN1* was markedly upregulated in cells on PA6 + GO2, whereas it was downregulated on PA6 and PA6 + GO1 relative to the glass control. *FAK* expression was elevated exclusively in cells cultured on GO1-coated PA6. Conversely, integrin subunits (*β1*, *α5*, *α2*) and *ACTβ* were generally downregulated across all PA6-based scaffolds compared to the glass control.

At first glance, the concurrent presence of robust colonization and viability alongside decreased integrins and *ACTβ* transcript levels may seem counterintuitive. However, this apparent dissociation can be elucidated within the framework of mechanotransduction. Integrin-based adhesions serve as mechanosensitive structures that translate extracellular cues, such as substrate stiffness and geometric constraints, into intracellular signalling through focal adhesions and associated pathways. The mechanisms underlying integrin-mediated mechanotransduction have been comprehensively reviewed in the literature [50].

Given its highly rigid and continuous two-dimensional structure, the glass control typically promotes extensive cell spreading, thereby increasing the baseline expression of mechanosensitive and cytoskeletal genes. In contrast, fibrous or porous PA6-based scaffolds offer a discontinuous contact interface and a distinct effective mechanical environment at the cellular scale, which can diminish the transcriptional demand for specific integrin subunits and cytoskeletal components at 24 h, even when functional attachment and cell viability are preserved [51]. Moreover, integrin signalling is heavily regulated at the protein level through activation state changes, clustering, recycling, and post-translational events, including phosphorylation of focal adhesion components [52]. Thus, reduced mRNA levels of specific integrin subunits do not necessarily indicate impaired adhesion capacity. This concept is supported by GO-biointerface analyses, which demonstrate that enhanced filopodia spreading and formation may occur concurrently with transient and/or heterogeneous regulation of integrin-related signals, reflecting dynamic mechanosensory adaptation [35]. In addition, the 24 h time point may represent a post-attachment adaptation phase rather than the early transcriptional peak of integrins expression, which can occur within the first hours after seeding. Within this context, the strong upregulation of *FN1* on PA6 + GO2 is particularly informative. Fibronectin is a key ECM protein that controls cell adhesion, spreading, and migration at the biomaterial interface and is strongly influenced by surface-driven protein adsorption processes and ECM remodeling processes [53]. Increased *FN1* expression indicates that cells on PA6 + GO2 actively engage in ECM deposition and/or remodeling, potentially creating a fibronectin-rich microenvironment that stabilizes adhesion and supports the elongated morphology observed by SEM (Figure 3A). This scenario is consistent with elevated *FAK* expression, as *FAK* functions as a central component of focal adhesions, integrating signals from adhesion complexes, and has been specifically recognized as a key regulator of mechanotransduction and force-dependent signaling [54]. Notably, mechanotransduction studies also demonstrate that lower *FAK* expression under certain mechanical contexts can associate with preserved or even enhanced growth-related outcomes, reinforcing that focal-adhesion signalling strength is not linearly proportional to viability or proliferation across all microenvironments [55].

Building upon existing knowledge of in vitro responses and surface characteristics, it is essential to elucidate how nanostructural modifications manifest in biological responses at the tissue level. Consequently, evaluating the interactions between GO-nanostructured scaffolds and living tissue is imperative for gaining a comprehensive understanding of their capacity to facilitate tissue regeneration and promote cellular differentiation in vivo. PA6 scaffolds nanostructured with GO supported the growth of primary connective tissue, cartilage, and bone derived from the chicken embryo *Gallus gallus*, as confirmed by SEM visualization after 7 days of tissue culture on the scaffolds (Figure 4). Overall, tissue cells cultured on PA6-GO2 exhibited greater morphological variability depending on the tissue type, displaying elongated or epithelial morphologies that may indicate tissue differentiation. In contrast, cells on PA6 and PA6 + GO1 scaffolds predominantly exhibited spherical shapes, suggesting a less differentiated state compared to those on PA6 + GO2. However, these hypotheses should be further verified by more advanced molecular analyses. Nevertheless, our observations are consistent with existing literature demonstrating the osteoinductive effects of GO and its capacity to stimulate cell differentiation within polymer-based composite scaffolds [56,57,58].

For example, enrichment of electrospun silk fibers with GO and BMP-2 polypeptide has the potential to upregulate the expression of genes associated with the differentiation of bone marrow mesenchymal stem cells (MSCs), including osteopontin (*OPN*), runt-related transcription factor 2 (*RUNX2*), and alkaline phosphatase (*ALP*) [58]. Similarly, the incorporation of GO into PLGA fibers may enhance the wettability of the nonwoven fabric and its capacity for protein adsorption, thereby promoting the upregulation of *RUNX2* and *OPN* gene expression in pro-osteoblasts, along with increased ALP activity [25]. Furthermore, the addition of GO and BMP-2 within PLGA fibers may facilitate the adsorption of other bioactive molecules, such as hydrocortisone and trans-resveratrol, which are known to influence osteogenesis and angiogenesis [59]. The integration of GO into polymer scaffolds may also promote hydroxyapatite (HA) deposition, with the extent of mineralization being directly proportional to the GO concentration in the polymer matrix. This is attributed to GO’s ability to absorb calcium ions (Ca^2+^), thereby serving as nucleation sites for calcium phosphate layer formation, which is essential for bone mineralization [60]. In addition, GO incorporation can modulate the physical properties of polymer nonwovens by reducing porosity, which enhances hydrophilicity and mechanical strength, thereby mimicking the native properties of bone tissue; these modifications can lead to improved osteogenic outcomes, even during the early stages of tissue development [61]. Moreover, GO addition has been observed to decrease the depth of valleys in polymer fibers and reduce parameters such as the arithmetic mean height (Sa) and root mean square height (Sq), which correlates with surface smoothing [62]. It also increases surface hardness, potentially fostering a microenvironment conducive to bone tissue regeneration.

## 4. Materials and Methods

### 4.1. Graphene Oxide

Graphene oxide (GO) hydrocolloid at a concentration of 4000 ppm was purchased from Advanced Graphene Products (Zielona Góra, Poland). Prior to use, the solutions were sonicated using a Cell™ Ultrasonic Liquid Processor (Sonics & Materials, Newtown, CT, USA) under the following conditions: 5 min, 30 s pulses, 20% amplitude.

Detailed characterization of GO, including stability in ultrapure water and cell culture media, polydispersity index, hydrodynamic diameter, flake size distribution, topography, as well as chemical bonds and functional groups, has been presented previously [35]. The morphology of GO flakes was assessed by transmission electron microscopy (TEM JEM-1220; JEOL, Tokyo, Japan) at 80 keV using an 11-megapixel Morada camera (Olympus Soft Imaging Solutions, Münster-Nord, Germany).

### 4.2. Cell Culture Model

The cellular model used in this study was the HS-5 cell line (human bone marrow stromal cells with fibroblast-like morphology), obtained from the American Type Culture Collection (ATCC, Manassas, VA, USA). The cells were maintained in high-glucose (4.5 g/L) Dulbecco’s Modified Eagle Medium (DMEM), supplemented with 10% fetal bovine serum (FBS), and 1% antibiotic–antimycotic solution containing: penicillin (100 U/mL), streptomycin (100 μg/mL), amphotericin B (250 ng/mL). All reagents were purchased from Gibco™ (Thermo Fisher Scientific, Waltham, MA, USA). The cultures were incubated at 37 °C in a humidified atmosphere (95%) with 5% CO_2_.

### 4.3. Nanofilm Preparation

GO colloids at fivefold concentrations of 50 ppm, 125 ppm, 250 ppm, 500 ppm, 750 ppm, and 1000 ppm were prepared by diluting the GO stock solution with ultrapure water. Subsequently, 20 μL or 500 μL of GO colloid was applied to 96-well and 6-well plates, respectively, resulting in final GO concentrations of 10 ppm, 25 ppm, 50 ppm, 100 ppm, 150 ppm, and 200 ppm. Four replicates were prepared for each GO concentration in the 96-well plates, while two replicates were prepared for the 25 ppm and 50 ppm concentrations in the 6-well plates. The plates were then dried under laminar flow conditions for two days to facilitate the formation of GO nanofilms at the specified concentrations.

### 4.4. Biological Effect of GO Nanofilms

#### 4.4.1. Viability Assessment–MTT Assay

A mitochondrial dehydrogenase activity assay was performed to evaluate the viability of HS-5 cells. This colorimetric method is based on the reduction of a yellow tetrazolium salt to purple formazan crystals by metabolically active cells. The intensity of the purple color is directly proportional to mitochondrial dehydrogenase activity and, therefore, to cell viability. Cells were seeded at a density of 1.5 × 10^5^ cells/mL, with 100 μL per well. After 24 h, 20 μL of tetrazolium salt (5 mg/mL in PBS; Sigma Aldrich, St. Louis, MO, USA) was added directly to each well, and the plates were incubated for an additional 3 h. The medium was then removed, and 100 μL of solubilization buffer (2% Triton X-100 in isopropanol acidified with HCl to a final concentration of 0.01 M) was added to each well. The plate was shaken for 10 min (200 rpm) to dissolve the formazan crystals, followed by centrifugation at 1200 rpm for 10 min. The supernatant was transferred to a new plate, and absorbance was measured at 570 nm with a reference wavelength of 630 nm using an Infinite M200 microplate reader (Tecan Group Ltd., Männedorf, Germany). Four replicates were performed for each group. Blank wells (cell-free controls) were included to subtract background absorbance.

#### 4.4.2. Cell Morphology Evaluation

To visualize the general morphology of HS-5 cells, May-Grünwald-Giemsa (MGG) staining was performed. A 6-well plate was prepared with dried GO nanofilms on microscope slides using two GO concentrations selected based on cytotoxicity assay results—25 ppm and 50 ppm.

HS-5 cells were seeded onto nanofilms at a density of 1 × 10^5^ cells/mL in a volume of 2 mL. After 24 h of incubation, the cells were washed with PBS and fixed with 4% paraformaldehyde (PFA) for 15 min at room temperature. Subsequently, the cells were washed three times with PBS, and 1 mL of May-Grünwald’s reagent (Carl Roth, Karlsruhe, Germany) was added. Following a 3 min incubation, 1 mL of PBS was added and incubated for an additional 3 min. Then, 0.5 mL of 10-fold diluted Giemsa stain (Carl Roth) was added, and the samples were incubated for 15 min. Finally, the stain was removed, and the samples were washed with PBS and distilled water. The morphology of the cells was examined under an inverted light microscope (Olympus CKX41, Olympus Corporation, Tokyo, Japan).

#### 4.4.3. Cell Ultrastructure Visualization

Scanning electron microscopy (SEM; Quanta 200, FEI, Hillsboro, OR, USA) was employed to visualize the ultrastructure and cellular growth on GO nanofilms at high resolution. Cells were maintained on the nanofilms as described for May-Grünwald-Giemsa (MGG) staining. Following incubation, the culture medium was removed, and the wells were washed with PBS. Cells were then fixed in preheated 2.5% glutaraldehyde (GA) and incubated at 4 °C overnight. Subsequently, the cells were washed three times with PBS and post-fixed in osmium tetroxide (OsO_4_) for 45 min at room temperature. After fixation, the osmium tetroxide was washed three times with PBS, and the samples were dehydrated through a series of ethanol concentrations: 25%, 50%, 60%, 70%, 80%, 90%, and 95% (5 min at each concentration), followed by two incubations in 99.9% ethanol (15 min each). Finally, the samples were sputter-coated with this gold layer and examined using an SEM.

### 4.5. Polyamide 6

#### 4.5.1. Cell Scaffold Fabrication—Nanospider Technology

The preparation methods are based on a methodology previously employed in the publication by Yalcinkaya B. et al. [63]. Polyamide 6 (PA6; BASF, Ludwigshafen, Germany) was dissolved in a mixture of acetic acid and formic acid (weight ratio of 2:1) resulting in a polymer final concentration of 8% (*w*/*w*). The polymer solution was heated to 80 °C and stirred for 4 h. Polymer fibers were produced using Nanospider NSLAB equipment (NS 1WS500U, Elmarco s.r.o., Liberec, Czech Republic), which employs a modified wire electrode electrospinning process. The process was conducted with the following parameters: voltage—55 kV (wire), −10 kV (collector); distance between electrodes—18 cm; carriage speed—245 mm/s; wire rotation—40.5 cm/h; collector speed—9 cm/min; inlet air temperature—23 °C; RH—30%; inlet/outlet flow—98/110 m^3^/h.

#### 4.5.2. Nanostructuring of PA6 with GO

To enhance the physicochemical properties and bioactivity of PA6, a composite consisting of PA6 and GO was developed. PA6 was cut into 22 mm × 22 mm fragments, and GO was deposited onto the surface; the GO concentration was determined based on prior biocompatibility assessments of GO nanolayers (50 ppm). The nanostructuring of PA6 with GO was performed using two methods: (1) shaking (overnight at 300 rpm) and (2) direct deposition onto the polymer surface. The samples were designated as PA6 + GO1 (shaking) and PA6 + GO2 (direct deposition). Following preparation, samples were dried at 40 °C for two days.

#### 4.5.3. Surface Morphology and Texture Characterization

The morphology of the samples was characterized via scanning electron microscopy (SEM). Prior to imaging, samples were sputter-coated with a thin gold layer. Quantitative analysis of polymer fiber diameter distribution was conducted using ImageJ (version 1.52a).

Confocal scanning is performed using the Neox technology patented by SENSOFAR (Barcelona, Spain). The microdisplay is based on ferroelectric liquid crystal on silicon (FLCoS), which is a fast-switching device with no moving parts, enabling very fast and stable confocal image scanning with virtually unlimited lifetime. The Neox system uses a high-resolution CMOS sensor with up to 1232 × 1028 pixels in combination with a display resolution of 2560 × 1440 pixels. Measurements are carried out using an integrated LED light source, providing real-time sample imaging while simultaneously enabling thin film measurements.

Surface roughness measurements were performed over an area of 850.08 µm × 709.32 µm in accordance with the standard ČSN EN ISO 25178-2 (014451), 2023. The evaluation of surface roughness was carried out using the following parameters: Sa (arithmetical mean height)—the average absolute height deviation of the surface from the mean plane; Sq (root mean square height)—the root mean square of height deviations; and Sv (maximum pit depth)—the depth of the deepest valley below the mean plane.

#### 4.5.4. Chemical Characterization of Materials

Fourier-transformed infrared spectroscopy (FT-IR) was conducted to assess the chemical bonds and functional groups, using an FT-IR spectrometer (Nicolet iS5, Thermo Fisher Scientific) equipped with a diamond crystal iD7 ATR (attenuated total reflection) accessory. The FT-IR spectrum of the samples was obtained within the wavenumber range of 4000 to 400 cm^−1^, maintaining a resolution of 4 cm^−1^. OMNIC 9 software (version 9.12.1002, driver version 9.13.1224, Thermo Fisher Scientific) was used to analyze the spectra.

#### 4.5.5. Mechanical Properties of Materials

The mechanical properties of PA6 and PA6 + GO were evaluated through tensile testing utilizing a CTX texture analyzer (AMETEK Brookfield, Middleboro, MA, USA). Samples were prepared as rectangular strips measuring 1 cm × 4 cm. For each material, the test was conducted in triplicate. The samples were stretched uniaxially at a steady strain rate of 0.5 mm/min. The stress–strain curves obtained from the tests were used to calculate the elongation at break normalized to sample thickness.

### 4.6. Biological Properties of the PA6 + GO Cell Scaffold

#### 4.6.1. Cell Culture on the PA6 + GO

Biomaterials were sterilized by immersion in 70% ethanol for 30 min, rinsed three times with ultrapure water for 10 min, and then dried at room temperature for two days before further studies. Materials were cut to a size of 0.9 cm^2^ to fit the wells of 8-chamber flexiPERM^®^ inserts (Sarstedt, Nümbrecht, Germany). Cells were seeded onto the materials in the inserts at a density of 2 × 10^5^ cells/mL in a volume of 0.5 mL per well, in eight replicates for each experiment.

#### 4.6.2. Viability Evaluation—Presto Blue Assay

The PrestoBlue assay (Invitrogen, Thermo Fisher Scientific, Carlsbad, CA, USA) was employed to evaluate the viability of HS-5 cells cultured on scaffolds. This assay relies on the reduction of resazurin by cellular metabolic activity—specifically, by metabolites such as NADH, NADPH, and FADH_2_—to generate the fluorescent compound resorufin. The reduction process results in a colorimetric shift from blue to pink and an increase in fluorescence, serving as an indicator of cell viability. Following a 24 h incubation of cells on the scaffold under standard culture conditions, 50 μL of PrestoBlue reagent was added to each well and incubated for an additional 4 h. Subsequently, 100 μL of the medium from each well was transferred to a 96-well plate in triplicate. Absorbance measurements were conducted at 570 nm, with a reference wavelength of 600 nm, while fluorescence was measured with excitation at 560 nm and emission at 590 nm, utilizing an Infinite M200 microplate reader.

#### 4.6.3. Cell Growth and Morphology on the Composite

After seeding the cells on PA6/GO composite, as described in Section 4.6.1, cells were incubated for 24 and 72 h in standard conditions, and then the samples were prepared for visualization on the SEM, as described earlier in Section 4.4.3.

#### 4.6.4. Gene Expression Analysis

Gene expression analysis was performed using real-time PCR to examine the expression of genes involved in cell adhesion. The target genes included: integrin α2 (*INGα2*), integrin α5 (*INGα5*), integrin β1 (*INGβ1*), focal adhesion kinase (*FAK*), fibronectin (*FN1*), and β-actin (*ACTβ*). Glyceraldehyde-3-phosphate dehydrogenase (*GAPDH*) was used as the reference gene. The primers (Genomed, Warsaw, Poland) used for the reaction are listed in Table 1.

After incubation, the culture medium was collected from each well, cells were rinsed with PBS and 200 μL of trypsin was added to detach the cells. Trypsin was neutralized with culture medium, and the contents were transferred to Falcon tubes. The suspended cells were centrifuged at 300× *g* for 5 min. The cells were washed twice with PBS, then the pellet was frozen in liquid nitrogen and stored at −80 °C until RNA isolation.

Total RNA was extracted using GeneMATRIX Universal RNA Purification Kit (E3598-02, EURx, Gdańsk, Poland), accordingly to the protocol, followed by elution in 30 μL of nuclease-free water. RNA purity and concentration were measured using a NanoDrop 2000 spectrophotometer (Thermo Fisher Scientific).

Complementary DNA (cDNA) was synthesized from the isolated RNA using the High-Capacity cDNA Reverse Transcription Kit (Applied Biosystems, Thermo Fisher Scientific). The concentration of synthesized cDNA was also quantified using the NanoDrop 2000.

The PCR reaction mixture contained 5 μL of PowerUp SYBR Green Master Mix (Applied Biosystems), 5 μL of cDNA template (100 ng in total), 3.5 μL of RNase-free water, 0.75 μL each of forward and reverse primers. A total of 15 μL of the reaction mixture was loaded into each well of a MicroAmp™ Fast Optical 48-Well Reaction Plate (Applied Biosystems). Each reaction was performed in triplicate for each group. Amplification was carried out using the StepOnePlus™ Real-Time PCR System (Thermo Fisher Scientific) with the following thermal cycling conditions: initial denaturation at 95 °C for 10 min, followed by 40 cycles of 95 °C for 15 s and 60 °C for 60 s.

#### 4.6.5. Tissue Development on Scaffolds

The biocompatibility of PA6 scaffolds was assessed at the tissue level using tissues collected from 9-day-old *Gallus gallus* (domestic chicken) embryos. The tissue model based on embryonic chicken cartilage and bone tissues was previously established, validated, and successfully applied by our research group, as described in our earlier publication [64]. Preparation procedures were performed under sterile conditions, following surface sterilization of the eggshell with a cotton swab soaked in 70% ethanol. After opening the eggshell, the embryo was carefully extracted and immediately decapitated. Connective tissue, bone, and cartilage were then meticulously isolated from the embryo. The tissue samples were then thoroughly rinsed with PBS to remove residual blood and tissue. Tissue fragments approximately 3–5 mm in diameter (connective tissue) or length (bone and cartilage) were dissected and carefully transferred to previously prepared PA6 scaffolds in a 6-well culture plate, with two replicates assigned for each tissue sample. Each well was supplemented with 2 mL of DMEM. Cultures were grown in a humidified incubator at 37 °C with 5% CO_2_ for 7 days. After the incubation period, samples were prepared for SEM visualization according to the protocol described previously in Section 4.4.3, with the exception of the osmium tetroxide fixation step.

### 4.7. Statistical Analysis

Statistical analysis and the significance of differences between experimental groups were assessed using one-way analysis of variance (ANOVA) followed by Dunnett’s post hoc test, performed in GraphPad Prism 10.4.1 (GraphPad Software, Boston, MA, USA). Differences were considered statistically significant at *p* < 0.05.

## 5. Conclusions

Nanostructuring PA6 with GO represents a strategic modification designed to mitigate the polymer’s inherent bioinertness through the facilitation of effective cell–material biointerface formation. The synergistic interaction between GO and the polymer substrate mitigates the individual limitations of each component while amplifying their respective advantageous properties, culminating in a composite biomaterial exhibiting improved biological compatibility and mechanical integrity. Bone marrow stromal cells showed increased adhesion to the nanostructured scaffold and upregulated fibronectin, a critical ECM protein that regulates cell mechanosensing, adhesion and spreading at the biomaterial interface. Additionally, the GO-enriched scaffold influences the overall architecture of developing connective tissue, bone, and cartilage. Consequently, nanostructuring with GO not only reduces the bioinertness of PA6 but also enhances cellular mechanosensitivity, thereby supporting more effective tissue development which has potential application in regenerative medicine applications. However, additional in vivo investigations are necessary to validate these findings. Combined with the scalable, highly reproducible, and industrially relevant Nanospider™ electrospinning manufacturing technology, this simple surface-engineering strategy offers a practical route toward the large-scale production and future clinical translation of nanostructured biomaterials.

## Figures and Tables

**Table 1:** Primers data for gene expression analysis on mRNA level by real-time ddCT method.

Gene	Forward Primer 5′ → 3′	Reverse Primer 5′ → 3′	Amplicon Size (bp)
*INGα2*	GGAACGGGACTTTCGCAT	GGTACTTCGGCTTTCTCATCA	154
*INGα5*	GCCGATTCACATCGCTCTCAAC	GTCTTCTCCACAGTCCAGCAAG	139
*INGb1*	GGATTCTCCAGAAGGTGGTTTCG	TGCCACCAAGTTTCCCATCTCC	143
*FAK*	CCCACCAGAGGAGTATGTCC	CCCAGGTCAGAGTTCAATAG	150
*FN1*	ACAACACCGAGGTGACTGAGAC	GGACACAACGATGCTTCCTGAG	143
*ACTβ*	GATGAGATTGGCATGGCTTT	GTCACCTTCACCGTTCCAGT	102
*GAPDH*	GAGAAGGCTGGGGCTCATTTG	CATGGTTCACACCCATG	97

## Data Availability

The original contributions presented in this study are included in the article. Further inquiries can be directed to the corresponding author.

## Abbreviations

The following abbreviations are used in this manuscript:

*ACTβ*	β-actin
ALP	Alkaline phosphatase
ANOVA	One-way analysis of variance
ATCC	American Type Culture Collection
cDNA	Complementary DNA
DMEM	Dulbecco’s Modified Eagle Medium
*FAK*	Focal adhesion kinase
FBS	Fetal bovine serum
*FN1*	Fibronectin
FT-IR	Fourier-transformed infrared spectroscopy
GA	Glutaraldehyde
*GAPDH*	Glyceraldehyde-3-phosphate dehydrogenase
GFNs	Graphene family nanomaterials
GO	Graphene oxide
HA	Hydroxyapatite
HS-5	Human bone marrow stromal cells
*INGs*	Integrins
MGG	May-Grünwald-Giemsa
MSCs	Mesenchymal stem cells
OPN	Osteopontin
OsO_4_	Osmium tetroxide
PA6	Polyamide 6
PCL	Polycaprolactone
PFA	Paraformaldehyde
PLGA	Poly(lactic-co-glycolic acid)
ROCK	Rho-associated kinase
RUNX2	Runt-related transcription factor 2
*Sa*	Arithmetical mean height
SEM	Scanning electron microscopy
*Sq*	Root mean square height
*Sv*	Maximum pit depth
TEM	Transmission electron microscopy
